# *Neisseria meningitidis* ST-11 Clonal Complex, Chile 2012

**DOI:** 10.3201/eid2102.140746

**Published:** 2015-02

**Authors:** Pamela Araya, Jorge Fernández, Felipe Del Canto, Mabel Seoane, Ana B. Ibarz-Pavón, Gisselle Barra, Paola Pidal, Janepsy Díaz, Juan C. Hormazábal, María T. Valenzuela

**Affiliations:** Instituto de Salud Pública de Chile, Santiago, Chile (P. Araya, J. Fernández, M. Seoane, G. Barra, P. Pidal, J. Díaz, J.C. Hormazábal, M.T. Valenzuela);; Universidad de Chile, Santiago (F. Del Canto);; Pan American Health Organization, Washington, DC, USA (A.B. Ibarz-Pavón)

**Keywords:** Neisseria meningitidis, bacteria, serogroup W, invasive meningococcal disease, ST-11, sequence type, hyperinvasive lineage, clonal complex, Chile

## Abstract

Serogroup W *Neisseria meningitidis* was the main cause of invasive meningococcal disease in Chile during 2012. The case-fatality rate for this disease was higher than in previous years. Genotyping of meningococci isolated from case-patients identified the hypervirulent lineage W:P1.5,2:ST-11, which contained allele 22 of the *fHbp* gene.

*Neisseria meningitidis* (meningococcus) is the causative pathogen of invasive meningococcal disease (IMD), which includes a set of infectious syndromes, mainly meningitis or meningococcemia (septicemia) and, less commonly, pneumonia or other infections (*1*). Humans are the only reservoir for meningococcus, which usually colonizes the upper respiratory tract of ≈8%–25% of persons (*1*).

In Chile, the incidence of IMD decreased steadily during 2000–2012 from 3.6 to 0.7 cases/100,000 inhabitants (*2*). However, deaths from this disease have not followed this trend; the case-fatality rate increased from 8.9% in 2009 to 14.1% in 2010, 14.7% in 2011, and 27.0% in 2012 (*2*,*3*). During this period, the distribution of meningoccal serogroups has changed. There has been a large increase in frequency of serogroup W meningococci, which has replaced serogroup B as the most common serogroup. 

A total of 101 cases, 57 culture-confirmed: 42/57 (73.7%) serogroup B and 1/57 (1.8%) serogroup W were reported in 2009; 78 cases: 56 culture-confirmed 36/56 (64.3%) serogroup B and 6/56 (10.7%) serogroup W were reported in 2010; 73 cases: 63 culture-confirmed, 32/63 (50.8%) serogroup B and 22/63 (34.9%) serogroup W were reported in 2011; and 133 cases: 103 culture-confirmed, 38/103 (36.9%) serogroup B and 60/103 (58.3%) serogroup W were reported in 2012 (*2*–*4*). We conducted this study to determine whether W meningococci belonged to a hypervirulent genetic lineage of the ST-11 clonal complex (CC).

## The Study

A national epidemiologic program for surveillance and control of IMD is conducted by the Department of Epidemiology of the Ministry of Health of Chile. Every national health care center must report suspected cases of IMD and send bacterial isolates to the Institute of Public Health of Chile (Santiago, Chile) or send cerebrospinal fluid samples when cultures have shown negative results. IMD cases are defined by clinical signs and symptoms (neck stiffness, altered state of consciousness, rash, meningeal irritation) and confirmed by isolation of *N. meningitidis* from cerebrospinal fluid, blood, or another sterile body fluid or tissue. Each case is coded according to the International Classification of diseases, 10th Revision, as meningitis (code A39.0), meningococcemia (A39.2), Waterhouse-Friderichsen syndrome (A39.1), other meningococcal infections (A39.8), and unspecified meningococcal infections (A39.9).

In 2012, a total of 32 health care centers located throughout Chile reported 133 IMD cases. Of these cases, 103 were laboratory confirmed by bacterial isolation and biochemical identification (*3*). Serogroup was determined by slide agglutination with polyclonal antibodies. Genosubtyping was conducted by amplifying and sequencing variable regions 1 and 2 of the *porA* gene as described by Russell et al. (*5*) Variants were defined by reviewing the *Neisseria PorA* typing database (http://pubmlst.org/neisseria/PorA/). Sequence types (STs) were determined as described by Maiden et al. (*6*) on the basis of housekeeping genes *abcZ*, *adk*, *aroE*, *fumC*, *gdh*, *pdh*, and *pgm*. Sequences were compared with those in the *Neisseria* locus/sequence definition database (http://pubmlst.org/), and STs and CCs were assigned. The nomenclature used in this report, when appropriate, is serogroup: genosubtype: CC.

The *fHbp* genetic variant was identified as described by Brehony et al. (*7*). Allele numbers were assigned by querying a public database (*Neisseria* Factor H binding protein sequence typing; http://pubmlst.org/neisseria/fHbp/). Genotyping with higher resolution was conducted by using pulsed-field gel electrophoresis (PFGE) and restriction endonuclease *Spe*I. Electrophoretic profiles were analyzed by using BioNumerics software (Applied Maths NV, Sint-Martens-Latem, Belgium). A PFGE pattern was considered unique when ≥1 DNA bands in the electrophoretic migration profile differed from each other. A code was assigned to each pattern. To establish the magnitude of associations, we cross-tabulated data and calculated odds ratios (ORs) by using Med Calc software version 12.4.0.0 (http://www.medcalc.org/). The 95% CI was established, and p values <0.05 were considered significant.

Serogroup B meningococci were isolated from 38 (36.9%) case-patients, and serogroup W meningococci were isolated from 60 (58.2%) case-patients (*4*). Serogroups C and Y meningococci were rarely isolated (3 and 2 isolates, respectively). Multilocus sequencing typing showed serogroup B isolates belonged mainly to ST-32 and ST-41/44 CCs, including 4 and 6 STs respectively. Of 60 W isolates, 98% belonged to ST-11 CC (Table 1). This finding is consistent with the serogroup of CCs found in W meningococci isolated in Chile during 2010 (4/4 W isolates analyzed) and 2011 (19/21 W isolates) (*8*). Among this CC, 3 STs were identified, of which ST-11 was the most common (Table 1).

**Table 1:** Clonal complexes and sequence types identified in main serogroups of *Neisseria meningitidis* isolated in Chile, 2012*

Serogroup, clonal complex	No. (%) isolates	Sequence type
B, n = 38		
ST-32	17 (44.7)	NA
	13	32
	2	3822
	1	7780
	1	9918
ST-41–44	20 (52.6)	NA
	14	44
	1	315
	1	8528
	2	9233
	1	9354
	1	10127
ST-461	1 (2.6)	NA
	1	461
W, n = 60		
ST-11	59 (98.3)	NA
	51	11
	7	1025
	1	2961
ST-22	1 (1.7)	NA
	1	184

*NA, not applicable.

Genosubtyping of W:ST-11 strains obtained during 2012 indicated that 58/60 strains belonged to genosubtype P1.5,2 (Table 2). In addition, sequence analysis of the *fHbp* gene identified allele 22 as the most common variant; it was present in 58 (96.7%) of 60 strains that belonged to the W serogroup (Table 2). Allele 22 of *fHbp* was not detected in strains belonging to other serogroups, which indicated a strong association with W meningococci. These results indicated that most IMD cases reported during 2012 in Chile were caused by a hypervirulent genetic lineage of *N. meningitidis* serogroup W.

**Table 2:** Characteristics of *Neisseria meningitidis* serogroup W isolates from 60 patients with invasive meningococcal disease, Chile, 2012*

Characteristic	Genotype				
	W:P1.5,2:ST-11, n = 58			W:P1.5,2–53:ST-11, n = 1	W:P1.18–1.3:ST-22, n = 1
Clinical outcome†	A39.0, n = 12	A39.2, n = 39	Other, n = 7	A39.2, n = 1	A39.0, n = 1
*fHbp* gene allele					
16, n = 1	0	0	0	0	1
19, n = 1	0	1	0	0	0
22, n = 58	12	38	7	1	0
PFGE pattern					
Cl-Nm-Spe-030, n = 7	1	5	1	0	0
Cl-Nm-Spe-031, n = 33	5	24	4	0	0
Cl-Nm-Spe-044, n = 2	0	2	0	0	0
Cl-Nm-Spe-046, n = 8	2	5	1	0	0
Cl-Nm-Spe-083, n = 1	0	0	0	0	1
Cl-Nm-Spe-084, n = 3	2	1	0	0	0
Cl-Nm-Spe-085, n = 2	1	1	0	0	0
Cl-Nm-Spe-086, n = 1	1	0	0	0	0
Cl-Nm-Spe-087, n = 1	0	0	1	0	0
Cl-Nm-Spe-088, n = 1	0	1	0	0	0
Cl-Nm-Spe-100, n = 1	0	0	0	1	0

*PFGE, pulsed-field gel electrophoresis. †Codes from International Classification of Diseases, 10th Revision. Clinical outcomes: meningitis (A39.0); meningococcemia (A39.2); Other: Waterhouse-Friderichsen syndrome (A39.1); meningococcemia unspecified (A39.4); other meningococcal infections (A39.8) and nonspecified invasive meningococcal disease (A39.9).

Serogroup W meningococci W:P1.5,2:ST-11 with allele 22 of *fHbp* were isolated from samples of 12 of 42 meningitis case-patients reported during 2012 and from 38 of 51 samples from meningococcemia case-patients. This association with meningococcemia cases was significant (OR 5.5, 95% CI 2.4–12.9, p = 0.0001). Overall, of 103 IMD case-patients in this study, 22 died. W:P1.5,2:ST-11 meningococci carrying allele 22 of *fHbp* were isolated from most patients with lethal cases. However, this association was not significant (16 of 22 deaths; OR 2.5, 95% CI 0.9–6.9, p = 0.08).

Analysis of W:P1.5,2:ST-11 meningococci by PFGE identified 9 electrophoretic patterns (Table 2). PFGE showed that most hypervirulent W:ST-11 clones were closely related to each other. Meningococci with a Cl-Nm-Spe-031 pattern caused most meningococcemia cases (Table 2). However, this association was not significant (OR 1.8, 95% CI 0.59–5.38, p = 0.31). Of 16 lethal cases reported that were associated with W meningococci, 9 were caused by strains with the Cl-Nm-Spe-031 pattern, 3 by strains with the Cl-Nm-Spe-030 pattern, 3 by strains with the Cl-Nm-Spe-046 pattern, and 1 by a strain with the Cl-Nm-Spe-085 pattern.

## Conclusions

We showed that most W meningococci belonged to a hypervirulent genetic lineage of the ST-11 CC. Hypervirulent serogroup W meningococci W:P1.5,2:ST-11, which has the *fHbp* gene allele 22, was the main cause of IMD in Chile during 2012. Its presence was associated with meningococcemia cases and partially accounted for more deaths during 2012 than in previous years. These isolates have a genetic profile similar to that of isolates from the first outbreak of IMD attributed to serogroup W, which affected the Hajj pilgrimage in Saudi Arabia in 2000, and to that of isolates from a larger outbreak in Burkina Faso in 2002 (*9*). However, these isolates from Chile have allele ID 22 of *fHbp* instead of alleles ID 9 or ID 23. This allele has also been found in a hypervirulent W:P1.5,2:ST-11 strain in Mali in 1994 (*10*), but we have not found more instances of its presence.

Some genotypes of these isolates have been detected in serogroup W strains obtained in previous years, specifically genosubtype (*8*), allele 22 of the *fHbp* gene (17 strains obtained in 2011), and specific PFGE patterns (9 strains were Cl-Nm-Spe-030, 4 were Cl-Nm-Spe-031, and 3 were Cl-Nm-Spe-046). These results indicate that hyperinvasive clones are circulating in Chile.
